# Supplementary figures and images for: Core genes involved in the regulation of acute lung injury and their association with COVID-19 and tumor progression: A bioinformatics and experimental study
Source: PLoS One. 2021 Nov 22;16(11):e0260450. doi: 10.1371/journal.pone.0260450 (PMC8608348; doi:10.1371/journal.pone.0260450)

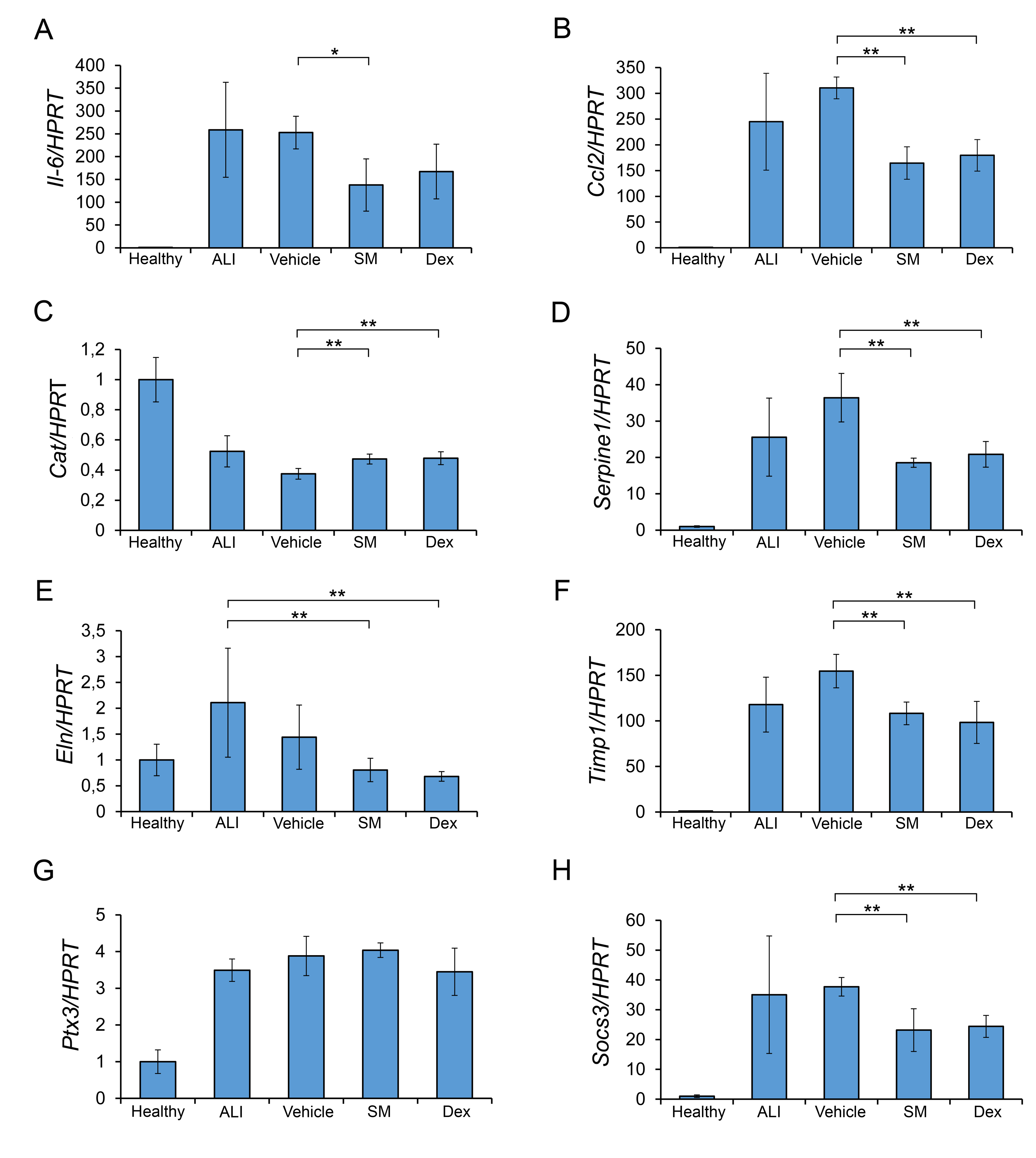

Supplement: S1 Fig — Data of qRT-PCR and TaqMan probes in the lung tissue of LPS-challenged mice without treatment and after SM and Dex administration. Relative expression levels were normalised to the level of hypoxanthine phosphoribosyltransferase (HPRT) (used as the reference gene). Three samples from each experimental group were analysed in triplicate. The data are shown as mean ± standard deviation. The statistical analysis was performed using the two-tailed unpaired t-test; p-values < 0.05 were considered as statistically significant. (TIF) [file pone.0260450.s001.tif]

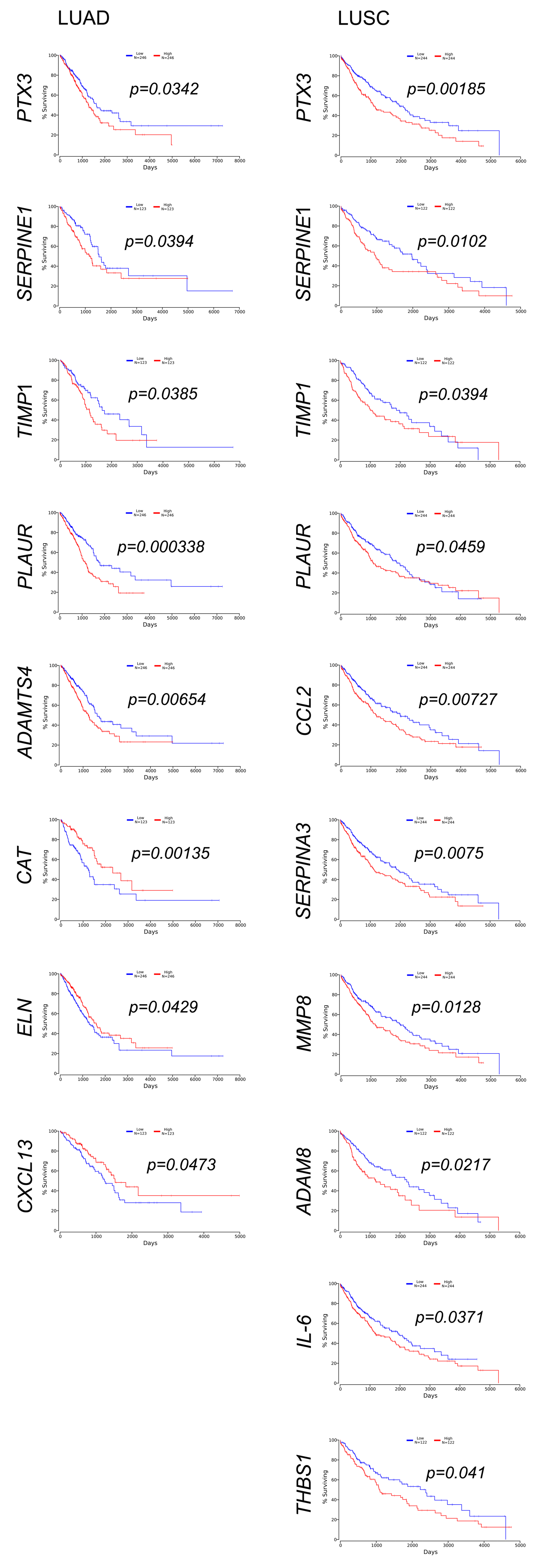

Supplement: S2 Fig — (TIF) [file pone.0260450.s002.tif]
