# Supplementary material for: Core genes involved in the regulation of acute lung injury and their association with COVID-19 and tumor progression: A bioinformatics and experimental study
Source: PLoS One. 2021 Nov 22;16(11):e0260450. doi: 10.1371/journal.pone.0260450 (PMC8608348; doi:10.1371/journal.pone.0260450)
Supplement: S1 Table — (DOCX) [file pone.0260450.s003.docx]

**S1 Table. Datasets used in the bioinformatics analysis.**

| **ID** | **Platform** | **Induction stimuli** | **Time after induction** | **Number of samples** | **Strain/**  **Cohort** | **Tissue** |
| --- | --- | --- | --- | --- | --- | --- |
| **Mice** | | | | | | |
| GSE130936 | Affymetrix Mouse Expression 430A Array | LPS | 22 h | 3 saline challenged mice;  4 LPS challenged mice | C57Bl/6J mice | Lung tissue |
| GSE80011 | Agilent Custom Mouse lncRNA (4*180K, Design ID:049801) | A/Wsn/33 Influenza virus | 24 h | 3 intact mice;  3 influenza infected mice | C57Bl/6J mice | Lung tissue |
| GSE94522 | Agilent-028005 SurePrint G3 Mouse GE 8x60K Microarray | Bleomycin | 24 h | 3 healthy mice;  3 bleomycin challenged mice | C57Bl/6J | Lung tissue |
| GSE58654 | Affymetrix Mouse Genome 430 2.0 Array | Hyperoxic injury | 24 h | 3 mice challenged with PBS, submitted to air inhalation;  3 mice challenged with PBS, challenged with 100% oxygen inhalation | ICR mice | Left lung tissue |
| **Human** | | | | | | |
| GSE21802 | GPL6102 Illumina human-6 v2.0 expression beadchip | Influenza virus H1N1 induced pneumonia | Day 1 after admission | 4 healthy samples;  6 influenza pneumonia samples without mechanical ventilation | Patients, attending to the intensive care unit (ICU) with primary viral pneumonia during the acute phase of influenza virus illness | Blood |
| GSE20346 | GPL6947 Illumina HumanHT-12 V.3.0 expression beadchip | Bacterial pneumonia | Day 1 after admission | 18 healthy samples;  6 bacterial pneumonia samples | Critically ill patients with severe infection and at least one major organ failure | Blood |
| GSE20346 | GPL6947 Illumina HumanHT-12 V.3.0 expression beadchip | Viral pneumonia | Day 1 after admission | 18 healthy samples;  4 viral pneumonia samples | Critically ill patients with severe infection and at least one major organ failure | Blood |
| GSE40012 | GPL6947 Illumina HumanHT-12 V.3.0 expression beadchip | Bacterial pneumonia | Day 1 after admission | 18 healthy samples;  16 bacterial pneumonia samples | Patients with severe community-acquired pneumonia requiring ICU admission | Blood |
| GSE40012 | GPL6947 Illumina HumanHT-12 V.3.0 expression beadchip | Viral pneumonia | Day 1 after admission | 18 healthy samples;  8 viral pneumonia samples | Patients with severe community-acquired pneumonia requiring ICU admission | Blood |
| GSE76293 | GLP570 [HG-U133_Plus_2] Affymetrix Human Genome U133 Plus 2.0 Array | Acute respiratory distress syndrome (ARDS) | Within 48 hours from diagnosis | 10 healthy samples;  12 ARDS samples | Patients, fulfilling the Berlin criteria for ARDS staying in mixed medical-surgical and neurosciences-trauma ICUs. | Blood polymorphonuclear neutrophils |
