# Supplementary material for: Core genes involved in the regulation of acute lung injury and their association with COVID-19 and tumor progression: A bioinformatics and experimental study
Source: PLoS One. 2021 Nov 22;16(11):e0260450. doi: 10.1371/journal.pone.0260450 (PMC8608348; doi:10.1371/journal.pone.0260450)
Supplement: S2 Table — (DOCX) [file pone.0260450.s004.docx]

**S2 Table. Association of ALI-related core genes with chronic lung pathology in human.**

| **Gene*** | **Chronic lung pathology** | | | | |
| --- | --- | --- | --- | --- | --- |
|  | **Idiopathic pulmonary fibrosis** | **COPD and chronic bronchitis** | **Bronchiectasis and cystic fibrosis** | **Emphysema** | **Systemic sclerosis** |
| *IL-6* | [1–3] | [4–7] | [8,9] |  | [10] |
| *CXCL13* | [11] | [12] | [13] | [14] | [15] |
| *TIMP1* | [16,17] | [18–20] | [21] | [22,23] |  |
| *CCL2* | [3,24] | [25,26] | [27] |  |  |
| *ADAM8* |  | [28,29] |  |  |  |
| *THBS1* | [30,31] | [32] |  |  |  |
| *SOCS3* | [1] | [33] | [34] |  |  |
| *SERPINA3* |  | [35] |  |  |  |
| *SERPINE1* | [1] | [36–38] |  |  |  |
| *PLAUR* |  | [38–40] | [39] |  | [41] |
| *TNC* | [42] | [43,44] |  |  | [45] |
| *MMP8* | [16] | [5,46,47] |  | [48] | [49] |
| *IFI44* | [50] |  |  |  | [51,52] |
| *RSAD2* |  | [53] |  |  |  |
| *PTX* |  |  | [54] | [55] | [56] |
| *LRG1* |  | [57] |  | [58] |  |
| *CAT* |  | [59–62] |  |  |  |
| *ELN* |  | [63] |  |  |  |

*Listed genes are involved in pathogenesis of mentioned chronic lung diseases, have prognostic value and correlate with the severity of the mentioned lung pathologies.

**References**

1. Xia Y, Lei C, Yang D, Luo H. Identification of key modules and hub genes associated with lung function in idiopathic pulmonary fibrosis. PeerJ. 2020;8. doi:10.7717/peerj.9848

2. Renaud L, da Silveira WA, Takamura N, Hardiman G, Feghali-Bostwick C. Prominence of IL6, IGF, TLR, and Bioenergetics Pathway Perturbation in Lung Tissues of Scleroderma Patients With Pulmonary Fibrosis. Front Immunol. 2020;11. doi:10.3389/fimmu.2020.00383

3. Fraser E, Denney L, Antanaviciute A, Blirando K, Vuppusetty C, Zheng Y, et al. Multi-Modal Characterization of Monocytes in Idiopathic Pulmonary Fibrosis Reveals a Primed Type I Interferon Immune Phenotype. Front Immunol. 2021;12. doi:10.3389/FIMMU.2021.623430

4. Zhang L, Sun Y. Muscle-Bone Crosstalk in Chronic Obstructive Pulmonary Disease. Front Endocrinol (Lausanne). 2021;12. doi:10.3389/FENDO.2021.724911

5. Dong T, Santos S, Yang Z, Yang S, Kirkhus NE. Sputum and salivary protein biomarkers and point-of-care biosensors for the management of COPD. Analyst. 2020;145: 1583–1604. doi:10.1039/C9AN01704F

6. Christenson SA. The role of genomic profiling in identifying molecular phenotypes in obstructive lung diseases. Current opinion in pulmonary medicine. Curr Opin Pulm Med; 2020. pp. 84–89. doi:10.1097/MCP.0000000000000646

7. Chung KF. Inflammatory mediators in chronic obstructive pulmonary disease. Current Drug Targets: Inflammation and Allergy. Curr Drug Targets Inflamm Allergy; 2005. pp. 619–625. doi:10.2174/156801005774912806

8. Dhooghe B, Noël S, Huaux F, Leal T. Lung inflammation in cystic fibrosis: Pathogenesis and novel therapies. Clin Biochem. 2014;47: 539–546. doi:10.1016/J.CLINBIOCHEM.2013.12.020

9. Shoki AH, Mayer-Hamblett N, Wilcox PG, Sin DD, Quon BS. Systematic review of blood biomarkers in cystic fibrosis pulmonary exacerbations. Chest. 2013;144: 1659–1670. doi:10.1378/chest.13-0693

10. Martinović Kaliterna D, Petrić M. Biomarkers of skin and lung fibrosis in systemic sclerosis. Expert Review of Clinical Immunology. Expert Rev Clin Immunol; 2019. pp. 1215–1223. doi:10.1080/1744666X.2020.1670062

11. Vuga LJ, Tedrow JR, Pandit K V., Tan J, Kass DJ, Xue J, et al. C-X-C motif chemokine 13 (CXCL13) is a prognostic biomarker of idiopathic pulmonary fibrosis. Am J Respir Crit Care Med. 2014;189: 966–974. doi:10.1164/rccm.201309-1592OC

12. Eide HA, Halvorsen AR, Sandhu V, Fåne A, Berg J, Haakensen VD, et al. Non-small cell lung cancer is characterised by a distinct inflammatory signature in serum compared with chronic obstructive pulmonary disease. Clin Transl Immunol. 2016;5: e109. doi:10.1038/CTI.2016.65

13. Frija-Masson J, Martin C, Regard L, Lothe M-N, Touqui L, Durand A, et al. Bacteria-driven peribronchial lymphoid neogenesis in bronchiectasis and cystic fibrosis. Eur Respir J. 2017;49. doi:10.1183/13993003.01873-2016

14. Faner R, Cruz T, Casserras T, Lopez-Giraldo A, Noell G, Coca I, et al. Network analysis of lung transcriptomics reveals a distinct b-cell signature in emphysema. Am J Respir Crit Care Med. 2016;193: 1242–1253. doi:10.1164/rccm.201507-1311OC

15. Taniguchi T, Miyagawa T, Toyama S, Yamashita T, Nakamura K, Saigusa R, et al. CXCL13 produced by macrophages due to Fli1 deficiency may contribute to the development of tissue fibrosis, vasculopathy and immune activation in systemic sclerosis. Exp Dermatol. 2018;27: 1030–1037. doi:10.1111/exd.13724

16. Todd JL, Vinisko R, Liu Y, Neely ML, Overton R, Flaherty KR, et al. Circulating matrix metalloproteinases and tissue metalloproteinase inhibitors in patients with idiopathic pulmonary fibrosis in the multicenter IPF-PRO Registry cohort. BMC Pulm Med. 2020;20. doi:10.1186/S12890-020-1103-4

17. ZhangHai-Tao, FangShen-Cun, WangCai-Ying, WangWei, WuJing, WangChun, et al. MMP-9 1562C>T Gene Polymorphism and Efficacy of Glucocorticoid Therapy in Idiopathic Pulmonary Fibrosis Patients. https://home.liebertpub.com/gtmb. 2015;19: 591–597. doi:10.1089/GTMB.2015.0057

18. Zhang Y, Li Y, Ye Z, Ma H. Expression of Matrix Metalloproteinase-2, Matrix Metalloproteinase-9, Tissue Inhibitor of Metalloproteinase-1, and Changes in Alveolar Septa in Patients with Chronic Obstructive Pulmonary Disease. Med Sci Monit. 2020;26: e925278. doi:10.12659/MSM.925278

19. Paci P, Fiscon G, Conte F, Licursi V, Morrow J, Hersh C, et al. Integrated transcriptomic correlation network analysis identifies COPD molecular determinants. Sci Rep. 2020;10. doi:10.1038/S41598-020-60228-7

20. Li H, Shi K, Zhao Y, Du J, Hu D, Liu Z. TIMP-1 and MMP-9 expressions in COPD patients complicated with spontaneous pneumothorax and their correlations with treatment outcomes. Pakistan J Med Sci. 2020;36: 192. doi:10.12669/PJMS.36.2.1244

21. Tarique AA, Sly PD, Cardenas DG, Luo L, Stow JL, Bell SC, et al. Differential expression of genes and receptors in monocytes from patients with cystic fibrosis. J Cyst Fibros. 2019;18: 342–348. doi:10.1016/j.jcf.2018.07.012

22. Xu L, Bian W, Gu X, Shen C. Differing Expression of Cytokines and Tumor Markers in Combined Pulmonary Fibrosis and Emphysema Compared to Emphysema and Pulmonary Fibrosis. http://dx.doi.org/101080/1541255520171278753. 2017;14: 245–250. doi:10.1080/15412555.2017.1278753

23. Zhou X-M, Hou G, Gu D-X, Wang Q-Y, Zhao L. Peroxisome proliferator-activated receptor-γ in induced sputum is correlated with MMP-9/TIMP-1 imbalance and formation of emphysema in COPD patients. J Thorac Dis. 2017;9: 3703. doi:10.21037/JTD.2017.09.10

24. Karman J, Wang J, Bodea C, Cao S, Levesque MC. Lung gene expression and single cell analyses reveal two subsets of idiopathic pulmonary fibrosis (IPF) patients associated with different pathogenic mechanisms. PLoS One. 2021;16. doi:10.1371/JOURNAL.PONE.0248889

25. Hao W, Li M, Pang Y, Du W, Huang X. Increased chemokines levels in patients with chronic obstructive pulmonary disease: Correlation with quantitative computed tomography metrics. Br J Radiol. 2021;94. doi:10.1259/bjr.20201030

26. Henrot P, Prevel R, Berger P, Dupin I. Chemokines in COPD: From implication to therapeutic use. International Journal of Molecular Sciences. Int J Mol Sci; 2019. doi:10.3390/ijms20112785

27. Hisert KB, Birkland TP, Schoenfelt KQ, Long ME, Grogan B, Carter S, et al. CFTR Modulator Therapy Enhances Peripheral Blood Monocyte Contributions to Immune Responses in People With Cystic Fibrosis. Front Pharmacol. 2020;11. doi:10.3389/fphar.2020.01219

28. Polverino F, Rojas-Quintero J, Wang X, Petersen H, Zhang L, Gai X, et al. A disintegrin and metalloproteinase domain-8: A novel protective proteinase in chronic obstructive pulmonary disease. Am J Respir Crit Care Med. 2018;198: 1254–1267. doi:10.1164/rccm.201707-1331OC

29. Oreo KM, Gibson PG, Simpson JL, Wood LG, Mcdonald VM, Baines KJ. Sputum ADAM8 expression is increased in severe asthma and COPD. Clin Exp Allergy. 2014;44: 342–352. doi:10.1111/cea.12223

30. Todd JL, Neely ML, Overton R, Durham K, Gulati M, Huang H, et al. Peripheral blood proteomic profiling of idiopathic pulmonary fibrosis biomarkers in the multicentre IPF-PRO Registry. Respir Res. 2019;20. doi:10.1186/s12931-019-1190-z

31. Kaiser R, Frantz C, Bals R, Wilkens H. The role of circulating thrombospondin-1 in patients with precapillary pulmonary hypertension. Respir Res. 2016;17. doi:10.1186/s12931-016-0412-x

32. Savarimuthu Francis SM, Larsen JE, Pavey SJ, Duhig EE, Clarke BE, Bowman R V., et al. Genes and gene ontologies common to airflow obstruction and emphysema in the lungs of patients with COPD. PLoS One. 2011;6. doi:10.1371/journal.pone.0017442

33. Springer J, Scholz FR, Peiser C, Dinh QT, Fischer A, Quarcoo D, et al. Transcriptional down-regulation of suppressor of cytokine signaling (SOCS)-3 in chronic obstructive pulmonary disease. J Occup Med Toxicol. 2013;8. doi:10.1186/1745-6673-8-29

34. Kopp BT, Fitch J, Jaramillo L, Shrestha CL, Robledo-Avila F, Zhang S, et al. Whole-blood transcriptomic responses to lumacaftor/ivacaftor therapy in cystic fibrosis. J Cyst Fibros. 2020;19: 245–254. doi:10.1016/j.jcf.2019.08.021

35. Kim SH, Ahn HS, Park JS, Yeom J, Yu J, Kim K, et al. A proteomics-based analysis of blood biomarkers for the diagnosis of COPD acute exacerbation. Int J COPD. 2021;16: 1497–1508. doi:10.2147/COPD.S308305

36. Wang H, Yang T, Li D, Wu Y, Zhang X, Pang C, et al. Elevated circulating PAI-1 levels are related to lung function decline, systemic inflammation, and small airway obstruction in chronic obstructive pulmonary disease. Int J COPD. 2016;11: 2369–2376. doi:10.2147/COPD.S107409

37. Waschki B, Watz H, Holz O, Magnussen H, Olejnicka B, Welte T, et al. Plasminogen activator inhibitor-1 is elevated in patients with COPD independent of metabolic and cardiovascular function. Int J COPD. 2017;12: 981–987. doi:10.2147/COPD.S128689

38. Jiang Y, Xiao W, Zhang Y, Xing Y. Urokinase-type plasminogen activator system and human cationic antimicrobial protein 18 in serum and induced sputum of patients with chronic obstructive pulmonary disease. Respirology. 2010;15: 939–946. doi:10.1111/j.1440-1843.2010.01799.x

39. Xiao W, Hsu YP, Ishizaka A, Kirikae T, Moss RB. Sputum cathelicidin, urokinase plasminogen activation system components, and cytokines discriminate cystic fibrosis, COPD, and asthma inflammation. Chest. 2005;128: 2316–2326. doi:10.1378/chest.128.4.2316

40. Huang Q, Xiong H, Shuai T, Wang Y, Zhang C, Zhang M, et al. The clinical value of suPAR in diagnosis and prediction for patients with chronic obstructive pulmonary disease: a systematic review and meta-analysis. Ther Adv Respir Dis. 2020;14. doi:10.1177/1753466620938546

41. Manetti M, Allanore Y, Revillod L, Fatini C, Guiducci S, Cuomo G, et al. A genetic variation located in the promoter region of the UPAR (CD87) gene is associated with the vascular complications of systemic sclerosis. Arthritis Rheum. 2011;63: 247–256. doi:10.1002/art.30101

42. Estany S, Vicens-Zygmunt V, Llatjós R, Montes A, Penín R, Escobar I, et al. Lung fibrotic tenascin-C upregulation is associated with other extracellular matrix proteins and induced by TGFβ1. BMC Pulm Med. 2014;14. doi:10.1186/1471-2466-14-120

43. López-Sánchez M, Muñoz-Esquerre M, Huertas D, Montes A, Molina-Molina M, Manresa F, et al. Inflammatory markers and circulating extracellular matrix proteins in patients with chronic obstructive pulmonary disease and left ventricular diastolic dysfunction. Clin Respir J. 2017;11: 859–866. doi:10.1111/crj.12428

44. Willis-Owen SAG, Thompson A, Kemp PR, Polkey MI, Cookson WOCM, Moffatt MF, et al. COPD is accompanied by co-ordinated transcriptional perturbation in the quadriceps affecting the mitochondria and extracellular matrix. Sci Rep. 2018;8. doi:10.1038/s41598-018-29789-6

45. Brissett M, Veraldi KL, Pilewski JM, Medsger TA, Feghali-Bostwick CA. Localized expression of tenascin in systemic sclerosis-associated pulmonary fibrosis and its regulation by insulin-like growth factor binding protein 3. Arthritis Rheum. 2012;64: 272–280. doi:10.1002/art.30647

46. Hu H, Cai C, Xue M, Luo J, Liao C, Huang H, et al. Increased MMP8 levels in atopic chronic obstructive pulmonary disease: A study testing multiple immune factors in atopic and non-atopic patients. Int J COPD. 2020;15: 1839–1848. doi:10.2147/COPD.S263313

47. Sng JHJ, Prazakova S, Thomas PS, Herbert C. MMP-8, MMP-9 and Neutrophil Elastase in Peripheral Blood and Exhaled Breath Condensate in COPD. COPD J Chronic Obstr Pulm Dis. 2017;14: 238–244. doi:10.1080/15412555.2016.1249790

48. Koo HK, Hong Y, Lim MN, Yim JJ, Kim WJ. Relationship between plasma matrix metalloproteinase levels, pulmonary function, bronchodilator response, and emphysema severity. Int J COPD. 2016;11: 1129–1137. doi:10.2147/COPD.S103281

49. Roderfeld M, Rath T, Schulz R, Seeger W, Tschuschner A, Graf J, et al. Serum matrix metalloproteinases in adult CF patients: Relation to pulmonary exacerbation. J Cyst Fibros. 2009;8: 338–347. doi:10.1016/j.jcf.2009.06.001

50. Rostami MR, Bradic M. The derepression of transposable elements in lung cells is associated with the inflammatory response and gene activation in idiopathic pulmonary fibrosis. Mob DNA. 2021;12. doi:10.1186/S13100-021-00241-3

51. Vlachogiannis NI, Pappa M, Ntouros PA, Nezos A, Mavragani CP, Souliotis VL, et al. Association Between DNA Damage Response, Fibrosis and Type I Interferon Signature in Systemic Sclerosis. Front Immunol. 2020;11. doi:10.3389/fimmu.2020.582401

52. Christmann RB, Sampaio-Barros P, Stifano G, Borges CL, De Carvalho CR, Kairalla R, et al. Association of interferon- and transforming growth factor β-regulated genes and macrophage activation with systemic sclerosis-related progressive lung fibrosis. Arthritis Rheumatol. 2014;66: 714–725. doi:10.1002/art.38288

53. Hilzendeger C, da Silva J, Henket M, Schleich F, Corhay JL, Kebadze T, et al. Reduced sputum expression of interferon-stimulated genes in severe COPD. Int J COPD. 2016;11: 1485–1494. doi:10.2147/COPD.S105948

54. Hamon Y, Jaillon S, Person C, Giniès JL, Garo E, Bottazzi B, et al. Proteolytic cleavage of the long pentraxin PTX3 in the airways of cystic fibrosis patients. Innate Immun. 2013;19: 611–622. doi:10.1177/1753425913476741

55. Zhang Y, Tedrow J, Nouraie M, Li X, Chandra D, Bon J, et al. Elevated plasma level of Pentraxin 3 is associated with emphysema and mortality in smokers. Thorax. 2021;76: 335–342. doi:10.1136/thoraxjnl-2020-215356

56. Iwata Y, Yoshizaki A, Ogawa F, Komura K, Hara T, Muroi E, et al. Increased serum pentraxin 3 in patients with systemic sclerosis. J Rheumatol. 2009;36: 976–983. doi:10.3899/jrheum.080343

57. Tan DBA, Ito J, Peters K, Livk A, Lipscombe RJ, Casey TM, et al. Protein Network Analysis Identifies Changes in the Level of Proteins Involved in Platelet Degranulation, Proteolysis and Cholesterol Metabolism Pathways in AECOPD Patients. COPD J Chronic Obstr Pulm Dis. 2020;17: 29–33. doi:10.1080/15412555.2019.1711035

58. Hisata S, Racanelli AC, Kermani P, Schreiner R, Houghton S, Palikuqi B, et al. Reversal of emphysema by restoration of pulmonary endothelial cells. J Exp Med. 2021;218. doi:10.1084/jem.20200938

59. Pérez-Peiró M, Martín-Ontiyuelo C, Rodó-Pi A, Piccari L, Admetlló M, Durán X, et al. Iron replacement and redox balance in non-anemic and mildly anemic iron deficiency copd patients: Insights from a clinical trial. Biomedicines. 2021;9. doi:10.3390/biomedicines9091191

60. Neves C, VKS L, LP L, MA M, ÉLM V, AL T, et al. Inflammatory and oxidative biomarkers as determinants of functional capacity in patients with COPD assessed by 6-min walk test-derived outcomes. Exp Gerontol. 2021;152. doi:10.1016/J.EXGER.2021.111456

61. Pandey S, Garg R, Kant S, Gaur P. Vitamin D, C-reactive protein, and oxidative stress markers in chronic obstructive pulmonary disease. Tzu Chi Med J. 2021;33: 80–86. doi:10.4103/tcmj.tcmj_198_19

62. Bel’skaya L V., Sarf EA, Solomatin D V., Kosenok VK. Salivary metabolic profile of patients with lung cancer, chronic obstructive pulmonary disease of varying severity and their comorbidity: A preliminary study. Diagnostics. 2020;10. doi:10.3390/diagnostics10121095

63. Brandsma CA, Van Den Berge M, Postma DS, Jonker MR, Brouwer S, Paré PD, et al. A large lung gene expression study identifying fibulin-5 as a novel player in tissue repair in COPD. Thorax. 2015;70: 21–32. doi:10.1136/thoraxjnl-2014-205091
